# Frequency of alterations in qSOFA, SIRS, MEWS and NEWS scores during the emergency department stay in infectious patients: a prospective study

**DOI:** 10.1186/s12245-021-00388-z

**Published:** 2021-11-27

**Authors:** Gideon H. P. Latten, Judith Polak, Audrey H. H. Merry, Jean W. M. Muris, Jan C. Ter Maaten, Tycho J. Olgers, Jochen W. L. Cals, Patricia M. Stassen

**Affiliations:** 1 Emergency Department, Zuyderland Medical Centre, Henri Dunantstraat 5, 6419 PC Heerlen, The Netherlands; 2 Zuyderland Academy, Zuyderland Medical Centre, Heerlen, The Netherlands; 3 Department of Family Medicine, Care and Public Health Research Institute, Maastricht University, Maastricht, The Netherlands; 4 Department of Internal Medicine, section acute internal medicine, University of Groningen, University Medical Centre Groningen, Groningen, The Netherlands; 5 Department of Internal Medicine, division general medicine, section acute medicine, Care and Public Health Research Institute, Maastricht University, Maastricht, The Netherlands

**Keywords:** Clinical rules, Infection, Emergency department

## Abstract

**Background:**

For emergency department (ED) patients with suspected infection, a vital sign-based clinical rule is often calculated shortly after the patient arrives. The clinical rule score (normal or abnormal) provides information about diagnosis and/or prognosis. Since vital signs vary over time, the clinical rule scores can change as well. In this prospective multicentre study, we investigate how often the scores of four frequently used clinical rules change during the ED stay of patients with suspected infection.

**Methods:**

Adult (≥ 18 years) patients with suspected infection were prospectively included in three Dutch EDs between March 2016 and December 2019. Vital signs were measured in 30-min intervals and the quick Sequential Organ Failure Assessment (qSOFA) score, the Systemic Inflammatory Response Syndrome (SIRS) criteria, the Modified Early Warning Score and the National Early Warning Score (NEWS) score were calculated. Using the established cut-off points, we analysed how often alterations in clinical rule scores occurred (i.e. switched from normal to abnormal or vice versa). In addition, we investigated which vital signs caused most alterations.

**Results:**

We included 1433 patients, of whom a clinical rule score changed once or more in 637 (44.5%) patients. In 6.7–17.5% (depending on the clinical rule) of patients with an initial negative clinical rule score, a positive score occurred later during ED stay. In over half (54.3–65.0%) of patients with an initial positive clinical rule score, the score became negative later on. The respiratory rate caused most (51.2%) alterations.

**Conclusion:**

After ED arrival, alterations in qSOFA, SIRS, MEWS and/or NEWS score are present in almost half of patients with suspected infection. The most contributing vital sign to these alterations was the respiratory rate. One in 6–15 patients displayed an abnormal clinical rule score after a normal initial score. Clinicians should be aware of the frequency of these alterations in clinical rule scores, as clinical rules are widely used for diagnosis and/or prognosis and the optimal moment of assessing them is unknown.

## Background

Measuring vital signs is indispensable when assessing patients with suspected infection in the emergency department (ED), as their values provide information on patients’ current disease status. Vital signs are often incorporated in clinical rules, which provide information on diagnosis and/or prognosis. Four well-known and frequently used clinical rules for medical patients in the ED are the quick Sequential Organ Failure Assessment (qSOFA) score, the Systemic Inflammatory Response Syndrome (SIRS) criteria, the Modified Early Warning Score and the National Early Warning Score (NEWS) [1–3].

In many EDs, a clinical rule score is calculated with a single set of vital signs, measured shortly after arrival. Depending on the ED’s protocol, a positive—or abnormal—score can have important implications, either by triggering specific treatment protocols (e.g. for sepsis in case of qSOFA and SIRS), or by prioritising patients in crowded settings. Although these protocols are all aimed at early detection of deteriorating patients, it is known that vital signs change during a patient’s ED stay, due to natural fluctuation, clinical deterioration, or improvement as a result of prehospital or ED treatment. It has not been investigated how often the scores of clinical rules change after a patient’s arrival in the ED. [4]

For physicians in the ED, it would be insightful to know the frequency of these changes, specifically taking cut-off points for treatment protocols or warning triggers for escalation of care into account. This information could be used to optimise monitoring, prioritisation and decision making.

In this prospective multicentre study, we therefore aim to investigate how often the scores of four frequently used clinical rules (qSOFA, SIRS, MEWS and NEWS) change during the ED stay of patients with suspected infection and which vital signs cause most alterations.

## Methods

### Design and setting

This prospective multicentre study included patients in three EDs in the Netherlands: Zuyderland Heerlen (large teaching hospital, > 30,000 ED visits/year), Maastricht University Medical Centre (MUMC+, university secondary and tertiary care teaching hospital, > 20,000 visits/year) and University Medical Centre Groningen (UMCG, university tertiary care teaching hospital, > 30,000 visits/year).

### Study population

Data were collected in three inclusion periods, based on the availability of research staff per inclusion site (centre 1: 26 March 2018–28 April 2018, centre 2: 25 June 2018–3 August 2018, centre 3: 2 March 2016–11 December 2019). Patients visiting the ED between 8 a.m. and 11 p.m. were screened for eligibility. We included adult patients (≥ 18 years), who presented to the ED with fever (≥3 8.0 °C) and/or suspected infection and who were able to provide informed consent. The clinical suspicion of infection was judged by the staff member on duty, either an emergency physician or internist acute medicine. The judgement was based on information provided by the referring physician and information available immediately after ED triage. Examples of signs suggestive of an infection included localised signs of an infection (e.g. erythema) or specific complaints (e.g. chills and/or coughing).

Participation in the study did not alter the treatment of patients, which was at the physician’s discretion. All three hospitals have a protocol for sepsis, which includes intravenous antibiotics, fluid resuscitation and oxygen supplementation.

The Institutional Review Boards of Zuyderland, The Maastricht University Medical Centre and The University Medical Centre Groningen ruled that the Dutch Medical Research Involving Human Subjects Act is not applicable and granted waivers (METCZ20180022, METC 2018-0420, METC 2015/164). All participants provided written informed consent. We used the Strengthening the Reporting of Observational Studies in Epidemiology guidelines for reporting this observational study [5].

### Data collection

For this study, we retrieved data on age, sex and—if assessed—triage urgency (determined using the Dutch version of the Manchester Triage System (MTS)) [6, 7]. In intervals of approximately 30 min during the patient’s ED stay (T0-T3), we measured the following six vital signs: blood pressure (mmHg), heart rate (beats per minute—bpm), respiratory rate (/min), level of consciousness (Glasgow Coma Scale, GCS), temperature (°C) and peripheral oxygen saturation (%). A complete set of vital signs was defined as measurement of all six parameters. A maximum of four sets were measured (T0-T3), depending on the patient’s length of ED stay. Patients with less than two complete sets were excluded from analysis, since it is not possible to investigate variation over time in these patients.

### Definitions

In order to improve clarity throughout the remainder of the manuscript, we provide some additional details on the definitions used. In this study, four vital-sign based *clinical rules* were investigated (SIRS, qSOFA, MEWS, NEWS) (Table 1). When the values of a patient’s vital signs are entered in one of these rules, they add up to a numeric value. Depending on the established cut-off points, a clinical rule can be normal (i.e. negative) or abnormal (i.e. positive). This is called the *clinical rule score*. As stated, these scores (normal or abnormal) can have important implications by triggering specific treatment protocols or by escalating care. Cut-off points for abnormal clinical rule scores were ≥ 2 points for qSOFA and SIRS, ≥ 4 points for MEWS and ≥ 5 points for NEWS [1–3].

**Table 1:** Clinical rules

Clinical rule	Included vital signs	Possible values	Normal score	Abnormal score
**qSOFA**	Respiratory rate Level of consciousness Systolic blood pressure	0–3 points	0–1 points	≥ 2 points
**SIRS**	Temperature Heart rate Respiratory rate White blood cell count	0–4 points	0–1 points	≥ 2 points
**MEWS**	Systolic blood pressure Heart rate Respiratory rate Temperature Level of consciousness	0–14 points	0–3 points	≥ 4 points
**NEWS**	Respiratory rate Oxygen saturation Supplemental oxygen Temperature Systolic blood pressure Heart rate Level of consciousness	0–20 points	0–4 points	≥ 5 points

*Abbreviations*: qSOFA, quick Sequential Organ Failure Assessment; SIRS, Systemic Inflammatory Response Syndrome; MEWS, Modified Early Warning Score; NEWS, National Early Warning Score

### Statistical analysis

Descriptive analyses were performed for age, sex, triage urgency and the values of the measured vital signs. We calculated the scores of qSOFA, SIRS, MEWS and NEWS and the corresponding clinical rule score (normal/abnormal) at the different intervals (T0-T3). We analysed how often the clinical rule score changed from normal to abnormal or vice versa, and we examined whether these alterations represented a switch from an abnormal to a normal score or from a normal to an abnormal score. In addition, we investigated the different patterns in clinical rule scores that occurred during the patients’ ED stay and analysed which vital signs caused most alterations. We specifically chose to perform analyses based on cut-off points, as these represent daily practice.

All statistical analyses were performed using IBM SPSS statistical software version 26 (Armonk, 2019). Continuous data were reported as means with standard deviation (SD) and compared using Students’ *T* test or as medians with interquartile ranges (IQR) and compared using the Mann-Whitney *U* test. We reported categorical data as absolute numbers and as valid percentages (to correct for missing data); they were compared using chi-square or Fisher exact tests. A *P* value < 0.05 was considered statistically significant.

Based on an expected proportion of patients in whom the qSOFA score changed from positive to negative or vice versa at least once being 15%, a desired precision of estimate of 2% and a confidence level of 95%, we found the minimum sample size to be 1225 participants. Since qSOFA is currently recommended as the bedside tool for identifying poor clinical outcome in patients with (suspected) infections, we used this clinical rule to calculate the required sample size [3].

## Results

### Patients and vital signs

In total, 1743 patients were included during the study period. In 1433 (82.2%) of these patients, at least two complete sets of vital signs were measured (Table 2). Only these patients were included for analysis. The median age was 63 (IQR 51–72) years and 58.3% were male. The majority (63.1%) of patients were assigned triage urgency yellow [7].

**Table 2:** Baseline patient characteristics

		*n*
Age (median, IQR)	63 (51–72)	1433
Male (*n*, %)	835 (58.3%)	1433
Time between first and last measurement—min (median, IQR)	158 (112–225)	1433
Number of complete sets^a^ (*n*, %)		1433
- 2	373 (26.0%)	
- 3	553 (38.6%)	
- 4	507 (35.4%)	
Triage urgency upon arrival at the ED (*n*, %)		1193
- Red	1 (0.1%)	
- Orange	233 (16.3%)	
- Yellow	904 (63.1%)	
- Green	55 (3.8%)	
Vital signs at ED arrival		
- Systolic blood pressure—mmHg (median, IQR)	125 (111–140)	1412
- Heart rate—bpm (median, IQR)	75 (65–85)	1412
- Respiratory rate—/min (median, IQR)	19 (16–24)	1275
- Glasgow Coma Scale (median, IQR)	15 (15–15)	1378
- Temperature—°C (median, IQR)	37.5 (36.5–38.3)	1341
- Peripheral oxygen saturation—% (median, IQR)	96 (95–98)	1404

^*^Data are presented as median (IQR), or *n* (%)

*Abbreviations*: min, minute; bpm, beats per minute; ED, emergency department

^a^Complete set: systolic blood pressure, heart rate, respiratory rate, Glasgow Coma Scale, temperature, peripheral oxygen saturation

### Alterations in clinical rule scores

In total, 637 (44.5%) patients experienced one or more alterations in the score of one of the clinical rules (Table 3). Least alterations were present in the qSOFA scores (11.2%), whereas SIRS scores altered most often. The total number of alterations was 1593, of which 882 (55.4%) represented an improvement in patient status (abnormal to normal score) and 711 (44.6%) a deterioration (normal to abnormal score). In all clinical rules, approximately half (53.3–57.3%) of alterations represented patient improvement.

**Table 3:** Alterations in clinical rule scores

	All rules	qSOFA	SIRS	MEWS	NEWS
Patients with no alterations	796 (55.5%)	1273 (88.9%)	1055 (73.6%)	1149 (80.2%)	1059 (73.9%)
Patients with ≥1 alteration^a^	637 (44.5%)	160 (11.2%)	378 (26.4%)	284 (19.8%)	374 (26.1%)
- Patients with 1 alteration		101 (7.0%)	292 (20.4%)	203 (14.2%)	240 (16.7%)
- Patients with 2 alterations		53 (3.7%)	75 (5.2%)	75 (5.2%)	120 (8.4%)
- Patients with 3 alterations		6 (0.4%)	11 (0.8%)	6 (0.4%)	14 (1.0%)
Total number of alterations	1593	225	475	371	522
- Switch from abnormal to normal	882 (55.4%)	120 (53.3%)	272 (57.3%)	211 (56.9%)	279 (53.4%)
- Switch from normal to abnormal	711 (44.6%)	105 (46.7%)	203 (42.7%)	160 (43.1%)	243 (46.6%)

^*^Data are presented as *n* (%)

^a^From normal to abnormal or vice versa

*Abbreviations*: qSOFA, quick Sequential Organ Failure Assessment; SIRS, Systemic Inflammatory Response Syndrome; MEWS, Modified Early Warning Score; NEWS, National Early Warning Score

### Patterns in clinical rule scores

Table 4 shows the different possible patterns of clinical rule scores during the patients’ ED stay. In the majority of patients, the first scores were normal (75.6–91.5%). In this group, most scores also remained normal during ED stay (82.5–93.3%). In 6.7–17.5%, however, an abnormal score occurred later on, representing a (temporary) deterioration of the patient.

**Table 4:**
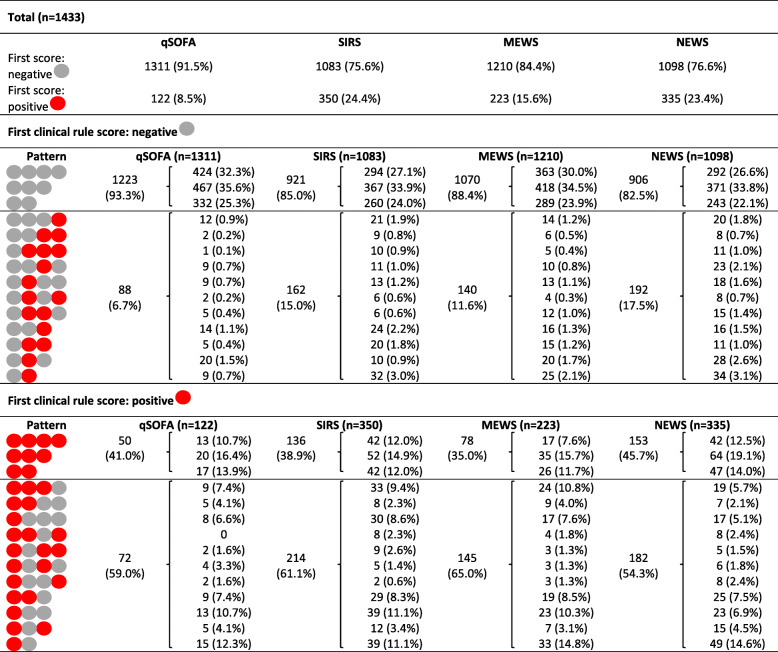
Patterns in clinical rule scores during emergency department stay

^*^Data are presented as *n* (%)

Legend:  negative clinical rule score,  positive clinical rule score

*Abbreviations*: qSOFA, quick Sequential Organ Failure Assessment; SIRS, Systemic Inflammatory Response Syndrome; MEWS, Modified Early Warning Score; NEWS, National Early Warning Score; ED, emergency department

Patients with an abnormal first clinical rule score had normal scores later on in 54.3–65.0%, representing a (temporary) improvement in patient status.

### Vital signs responsible for alterations in clinical rule scores

Table 5 shows which vital signs were responsible for the alterations in clinical rule scores. The respiratory rate was responsible for most alterations in all 4 clinical rules: 55.6% for qSOFA, 45.5% for SIRS, 50.9% for MEWS and 54.4% for NEWS. The least contributing vital sign for alterations in clinical rule scores was the level of consciousness (1.1–17.0%).

**Table 5:** Vital signs responsible for alterations in clinical rule scores

Clinical rule	Alteration**	***n***	Responsible vital sign(s)***					
			SBP	HR	RR	GCS	T	SpO_2_
**qSOFA**	Deterioration	105	59 (56.2%)		52 (49.5%)	15 (14.4%)		
	Improvement	120	58 (48.3%)		73 (60.8%)	23 (19.3%)		
	**Total**	**225**	**117 (52.0%)**		**125 (55.6%)**	**38 (17.0%)**		
**SIRS**	Deterioration	203		61 (30.0%)	98 (48.3%)		83 (40.9%)	
	Improvement	272		82 (30.1%)	118 (43.3%)		132 (48.5%)	
	**Total**	**475**		**143 (30.1%)**	**216 (45.5%)**		**215 (45.3%)**	
**MEWS**	Deterioration	160	39 (24.4%)	52 (32.5%)	87 (54.4%)	1 (0.6%)	52 (32.5%)	
	Improvement	211	40 (19.0%)	62 (29.4%)	102 (48.3%)	3 (1.4%)	107 (50.7%)	
	**Total**	**371**	**79 (21.3%)**	**114 (30.7%)**	**189 (50.9%)**	**4 (1.1%)**	**159 (42.9%)**	
**NEWS**	Deterioration	243	95 (39.1%)	67 (27.6%)	135 (55.6%)	4 (1.6%)	43 (17.7%)	117 (48.1%)
	Improvement	279	113 (40.5%)	84 (30.1%)	149 (53.4%)	4 (1.4%)	80 (28.7%)	143 (51.3%)
	**Total**	**522**	**208 (39.8%)**	**151 (28.9%)**	**284 (54.4%)**	**8 (1.5%)**	**123 (23.6%)**	**260 (49.8%)**

^*^Data are presented as *n* (%)

^**^Deterioration: change from normal to abnormal score, Improvement: change from abnormal to normal score

^***^Sum of percentages can exceed 100% as more than 1 vital sign could contribute to a change in the clinical rule score

*Abbreviations*: qSOFA, quick Sequential Organ Failure Assessment; SIRS, Systemic Inflammatory Response Syndrome; MEWS, Modified Early Warning Score; NEWS, National Early Warning Score; SBP, systolic blood pressure; HR, heart rate; RR, respiratory rate; GCS, Glasgow Coma Scale; T, temperature; SpO_2_, peripheral oxygen saturation

## Discussion

In this study, we investigated the frequency of alterations in qSOFA, SIRS, MEWS and NEWS scores in 1433 patients with suspected infection during their ED stay. We showed that qSOFA alterations were present in 1 in 9 patients, SIRS in 1 in 4, MEWS in 1 in 5 and NEWS in 1 in 4. Approximately half of alterations were from a normal to an abnormal score and half vice versa. Interestingly, 6.7–17.5% of patients with an initially normal clinical rule score turned abnormal later on, while over 50% of patients with an abnormal first score turned normal later on. The respiratory rate was responsible for over half of the changes in clinical rule scores.

To our knowledge, our study is the first to investigate the effect of vital sign variation in the ED on the scores of qSOFA, SIRS, MEWS and NEWS. Even during a relatively short median ED stay of 158 min, the clinical rule score changed in 11–26% of patients. The exploration of the progression of these clinical rule scores over time is a unique feature. In contrast, most ED-based studies use a single set of vital signs, either the first or the worst values, which may provide an explanation for the known suboptimal performance of many diagnostic and prognostic clinical rules in the ED. [8–10] When using clinical rules to predict poor outcome (like sepsis), repeated measurements of vital signs can be of surplus value, since the optimal moment of assessing clinical rule scores is unknown.

It is reassuring that the over half of patients with an abnormal score at arrival turned normal during their ED stay. Possible explanations for the improvement in vital signs include adequate response to treatment and regression towards the mean. Previous studies have shown similar results: patients with sepsis tend to improve during the first 3 h in the ED. [4] It is also known, however, that approximately one third of admitted medical patients with normal initial vital signs deteriorate within 24 h [11]. Depending on the clinical rule used, between one in 6–15 of our patients turned from normal to abnormal during their ED stay. Actual deterioration of these patients is the most likely explanation for this phenomenon. Since changes in vital signs can be subtle, it is not unlikely that gradual deterioration can be missed when vital signs are not measured on a regular basis. Although the clinical value of this finding has yet to be established, the potential value of repeated measurements has to be weighed against the time-consumption when performed manually or the background noise possibly created with automated or continuous measurements.

Worth mentioning as well is that a strong acute care chain is present in the Netherlands. Most ED patients are referred by a general practitioner (GP), and there is an important role for the highly trained emergency medical services (EMS) nurses [12, 13]. These professionals often initiate therapy (e.g. oxygen or fluid therapy) even before a patient arrives at the ED. As a result, vital signs may (temporarily) improve during a patient’s prehospital journey. The first values measured in the ED may therefore be better than those measured at home by the GP, potentially underestimating a patient's severity of illness upon arrival in the ED. Therefore, it must be recognised that measurements taken in the ED are not ‘the first measurements’. It is plausible that repeated measurements and adequate communication throughout the entire acute care chain can help optimise the care for these patients.

An interesting finding is that over half of all alterations in clinical rule scores could (partially or entirely) be attributed to variations in respiratory rate. The predictive value of the respiratory rate has long since been recognised, but the fact that it is usually measured manually reduces both the frequency and reliability of its measurements [14–18]. One could imagine that repeated manual measurements of respiratory rates in busy EDs are (too) labour intensive. We therefore feel that future research should investigate the reliability and value of non-invasive methods of either repeatedly or continuously measured respiratory rates.

Despite being the first to investigate the effect of vital sign variation in the ED on the scores of qSOFA, SIRS, MEWS and NEWS, our study has some limitations. First, the majority (63.1%) of our patients were triaged as MTS urgency yellow (‘urgent’). As a result, generalisation of the results to other populations should be done carefully. We would like to stress, however, that this is likely the group of patients that could most benefit from repeated measurements. Patients who are triaged as urgency red (‘immediately’) or orange (‘very urgent’) are acknowledged as having acute life-threatening problems and are usually assessed (almost) immediately by a physician, whereas ‘yellow’ patients have to be assessed within 1 h. In this hour, unwanted delay can occur. A second limitation is that we did not take therapeutic interventions and patient outcomes into account. Conclusions on what changes vital signs (and clinical rule scores) and whether our reported alterations are associated with adverse outcomes, such as intensive care admission or mortality, can therefore not be drawn. A hypothetical study taking all this into account would be labour-intensive, as not only interventions performed in the ED would have to be registered, but also prehospital interventions.

We feel that future research should focus on the feasibility, implementation and predictive value of repeated or continuous measurement of vital signs, throughout the acute care chain. Specific focus should lie on the respiratory rate, as it has been repeatedly shown to be an important predictor of clinical deterioration, but measured infrequently and inadequately as well.

## Conclusion

Almost half of patients with a suspected infection experience a change in the score of qSOFA, SIRS, MEWS and/or NEWS during ED stay. Approximately half of alterations were from a normal to an abnormal score and half vice versa. The respiratory rate was the most contributing vital sign to these alterations. Patients with a normal score at ED arrival had a 6.7–17.6% chance of displaying an abnormal score later during their ED stay, whereas 50% of patients with an initial abnormal score turned normal later on. Clinicians should be aware of the frequency of alterations in clinical rule scores and realise that the optimal moment of assessing clinical rule scores is unknown.

## Data Availability

The datasets used and/or analysed during the current study are available from the corresponding author on reasonable request.

## References

[1] Subbe CP, Kruger M, Rutherford P, Gemmel L (2001). Validation of a modified Early Warning Score in medical admissions. QJM.

[2] Smith GB, Prytherch DR, Meredith P, Schmidt PE, Featherstone PI (2013). The ability of the National Early Warning Score (NEWS) to discriminate patients at risk of early cardiac arrest, unanticipated intensive care unit admission, and death. Resuscitation.

[3] Singer M, Deutschman CS, Seymour CW, Shankar-Hari M, Annane D, Bauer M, Bellomo R, Bernard GR, Chiche JD, Coopersmith CM, Hotchkiss RS, Levy MM, Marshall JC, Martin GS, Opal SM, Rubenfeld GD, van der Poll T, Vincent JL, Angus DC (2016). The Third International Consensus Definitions for Sepsis and Septic Shock (Sepsis-3). JAMA.

[4] Quinten VM, van Meurs M, Ter Maaten JC, Ligtenberg JJ (2016). Trends in vital signs and routine biomarkers in patients with sepsis during resuscitation in the emergency department: a prospective observational pilot study. BMJ Open.

[5] von Elm E, Altman DG, Egger M, Pocock SJ, Gotzsche PC, Vandenbroucke JP (2014). The Strengthening the Reporting of Observational Studies in Epidemiology (STROBE) Statement: guidelines for reporting observational studies. Int J Surg.

[6] Mackway-Jones K, Marsden J, Windle J. Emergency triage. 3rd ed. Oxford: Wiley Blackwell; 2014.

[7] Manchester Triage Group. Triage voor de Spoedeisende Hulp. 3rd ed. Oxford: Bohn Stafleu van Loghum; 2016.

[8] Tusgul S, Carron PN, Yersin B, Calandra T, Dami F (2017). Low sensitivity of qSOFA, SIRS criteria and sepsis definition to identify infected patients at risk of complication in the prehospital setting and at the emergency department triage. Scand J Trauma Resusc Emerg Med.

[9] van der Woude SW, van Doormaal FF, Hutten BA, J Nellen F, Holleman F. (2018). Classifying sepsis patients in the emergency department using SIRS, qSOFA or MEWS. Neth J Med.

[10] Churpek MM, Snyder A, Han X, Sokol S, Pettit N, Howell MD, Edelson DP (2017). Quick sepsis-related organ failure assessment, systemic inflammatory response syndrome, and early warning scores for detecting clinical deterioration in infected patients outside the intensive care unit. Am J Respir Crit Care Med.

[11] Henriksen DP, Brabrand M, Lassen AT (2014). Prognosis and risk factors for deterioration in patients admitted to a medical emergency department. PLoS One.

[12] Latten GHP, Claassen L, Jonk M, Cals JWL, Muris JWM, Stassen PM (2019). Characteristics of the prehospital phase of adult emergency department patients with an infection: a prospective pilot study. PLoS One.

[13] Smits M, Rutten M, Keizer E, Wensing M, Westert G, Giesen P (2017). The development and performance of after-hours primary care in the Netherlands: a narrative review. Ann Intern Med.

[14] Latten GHP, Spek M, Muris JWM, Cals JWL, Stassen PM (2019). Accuracy and interobserver-agreement of respiratory rate measurements by healthcare professionals, and its effect on the outcomes of clinical prediction/diagnostic rules. PLoS One.

[15] Fieselmann JF, Hendryx MS, Helms CM, Wakefield DS (1993). Respiratory rate predicts cardiopulmonary arrest for internal medicine inpatients. J Gen Intern Med.

[16] Flenady T, Dwyer T, Applegarth J (2017). Explaining transgression in respiratory rate observation methods in the emergency department: a classic grounded theory analysis. Int J Nurs Stud.

[17] Leuvan CH, Mitchell I (2008). Missed opportunities? An observational study of vital sign measurements. Crit Care Resusc.

[18] Semler MW, Stover DG, Copland AP, Hong G, Johnson MJ, Kriss MS, Otepka H, Wang L, Christman BW, Rice TW (2013). Flash mob research: a single-day, multicenter, resident-directed study of respiratory rate. Chest.

